# Amiodarone-related keratopathy and optic neuropathy: case report and literature review

**DOI:** 10.3389/fmed.2025.1572461

**Published:** 2025-04-02

**Authors:** Nana Meng, Leizhou Xia, Yiqing Gong, Peirong Lu

**Affiliations:** ^1^Department of Ophthalmology, The First Affiliated Hospital of Soochow University, Suzhou, China; ^2^Department of Ophthalmology, Affiliated People's Hospital, Jiangsu University, Zhenjiang, China; ^3^Zhenjiang Kangfu Eye Hospital, Zhenjiang, China; ^4^Department of General Surgery, Affiliated People's Hospital, Jiangsu University, Zhenjiang, China

**Keywords:** amiodarone, optic neuropathy, keratopathy, amiodarone-associated optic neuropathy, anterior ischemic optic neuropathy

## Abstract

Amiodarone, a highly effective yet lipophilic antiarrhythmic drug with prolonged half-life, is associated with systemic and ocular complications. While keratopathy being the most prevalent, affecting 70–100% of long-term users, amiodarone-associated optic neuropathy (AAON), though rare (incidence: 0.36–2%), can induce diverse visual impairments, ranging from mild deficits to profound vision loss. Given that patients on amiodarone frequently possess the risk factors of vascular diseases, it is essential to differentiate the diagnosis of AAON from non-arteritic anterior ischemic optic neuropathy (AION). This study reports a 61-year-old man who developed both corneal deposition and optic neuropathy during systemic amiodarone therapy. We further analyze the clinical features of keratopathy and optic neuropathy caused by amiodarone through a comprehensive literature review, aiming to enhance diagnostic recognition and management strategies.

## Introduction

Amiodarone is a class III antiarrhythmic drug with properties of class I, II, and IV, commonly used to treat ventricular and supraventricular arrhythmias (1). The compound exhibits oral bioavailability between 30 and 80%, with a half-life spanning 20 to 100 days (2). Amiodarone’s lipophilicity and strong tissue affinity contribute to its widespread toxicity, impacting various organs such as the lungs, thyroid, liver, skin, and nervous system, particularly with prolonged or high-dose usage (3, 4). Moreover, ocular side effects have been noted including keratopathy, lens opacities, retinopathy and optic neuropathy, among which the most common is vortex keratopathy, occurring in 70–100% of the patients (5). This form of keratopathy is typically benign, potentially reversible, and generally does not result in visual impairment. Amiodarone-associated optic neuropathy (AAON) is a relatively rare condition, with an incidence rate ranging from 0.36 to 2%. It has the potential to cause visual impairment, which can vary from mild to severe permanent vision loss (6). Several case reports and case series have linked the use of amiodarone to optic neuropathy or keratopathy (7, 8). However, there are relatively few reports documenting the co-occurrence of optic neuropathy and keratopathy in previous studies (9, 10). We present a case of a 61-year-old male with a history of ventricular tachycardia treated with amiodarone, who developed concurrent corneal and optic neuropathy in both eyes.

## Case presentation

In November 2023, a 61-year-old male patient was admitted to our hospital presenting with a complaint of bilateral blurred vision persisting for over 20 days. The patient had a four-year history of hypertension treated with metoprolol and a two-year history of hypertrophic non-obstructive cardiomyopathy without specific treatment. Four months ago, the patient received a dual-chamber implantable cardioverter-defibrillator (ICD) for persistent ventricular tachycardia, after which he began taking 200 mg of amiodarone daily to reduce the occurrence of ventricular tachycardia. The patient reported no history of systemic or ocular diseases and no history of ocular trauma.

**Figure 1:**
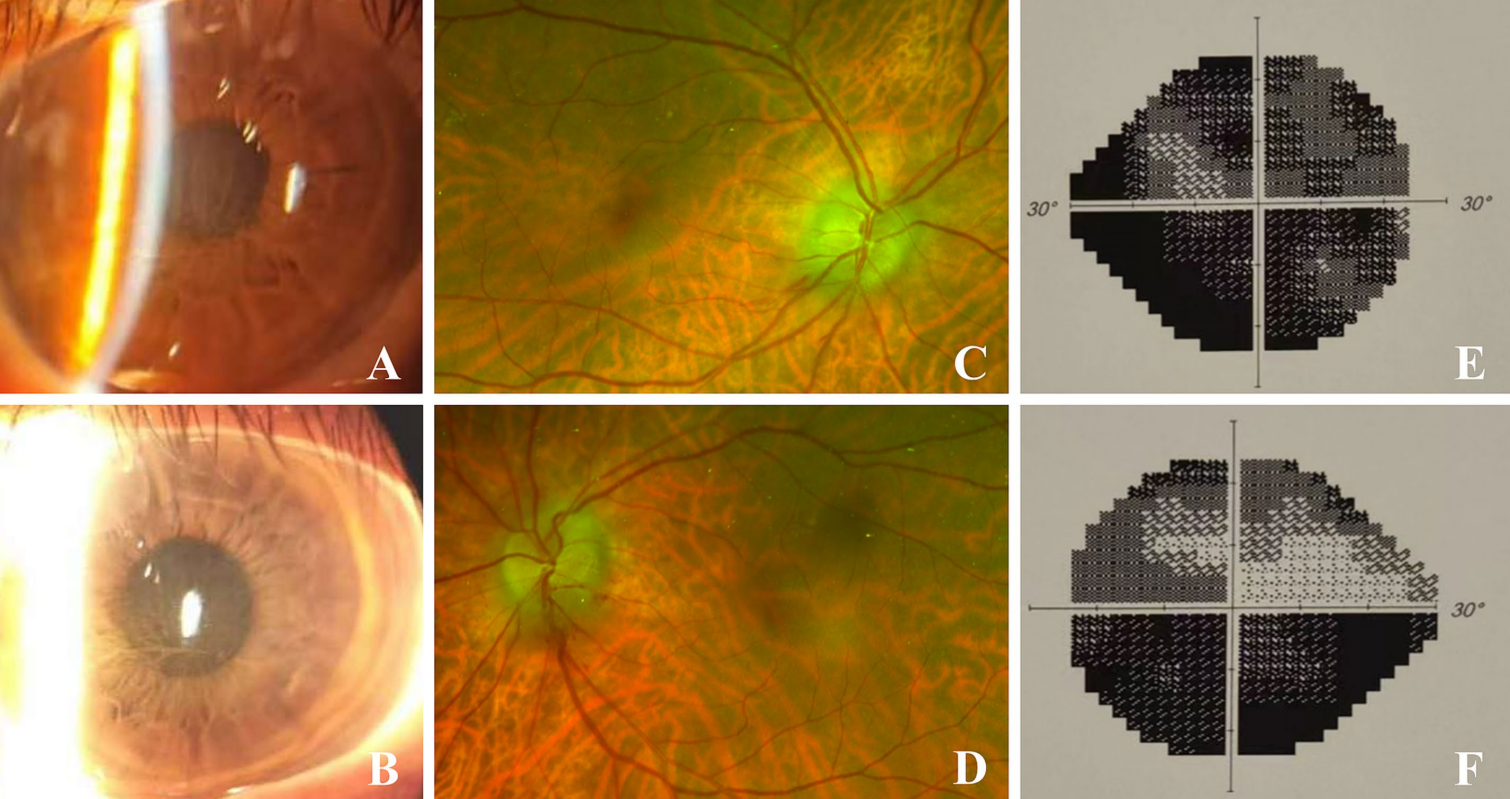
Binocular images on initial presentation. **(A,B)** were slit-lamp images of the right and left eyes respectively, showing greyish brown corneal epithelial opacities. **(C,D)** were fundus images of the right and left eyes respectively, showing disc edema and peridisk bleeding. **(E,F)** showed binocular perimetry indicating diffuse total defect in the right eye, inferior altitudinal defect and upper arcuate blind spots in the left eye.

A comprehensive ophthalmologic examination was conducted, encompassing best-corrected visual acuity (BCVA), slit-lamp examination, applanation tonometry, perimetry, and fundoscopy. Utilizing the international standard visual acuity chart, the BCVA was determined to be 0.08 for the right eye and 0.5 for the left eye. Slit-lamp examination revealed bilateral, symmetric, whorl-like brown deposits in the inferocentral cornea, with no fluorescein staining observed (Figures 1A,B). Intraocular pressures were normal, and mild lens opacities were noted in both eyes.

The pupil measures approximately 3.5 mm in diameter and exhibits a slow response to both direct and indirect light, with no relative afferent pupillary defect (RAPD) observed. The fundus examination revealed bilateral optic disc edema accompanied by peripapillary hemorrhages (Figures 1C,D), while no significant abnormalities were detected in the macular region. Perimetry revealed a diffuse defect in the right eye and inferior altitudinal as well as superior arcuate defects in the left eye (Figures 1E,F).

**Figure 2:**
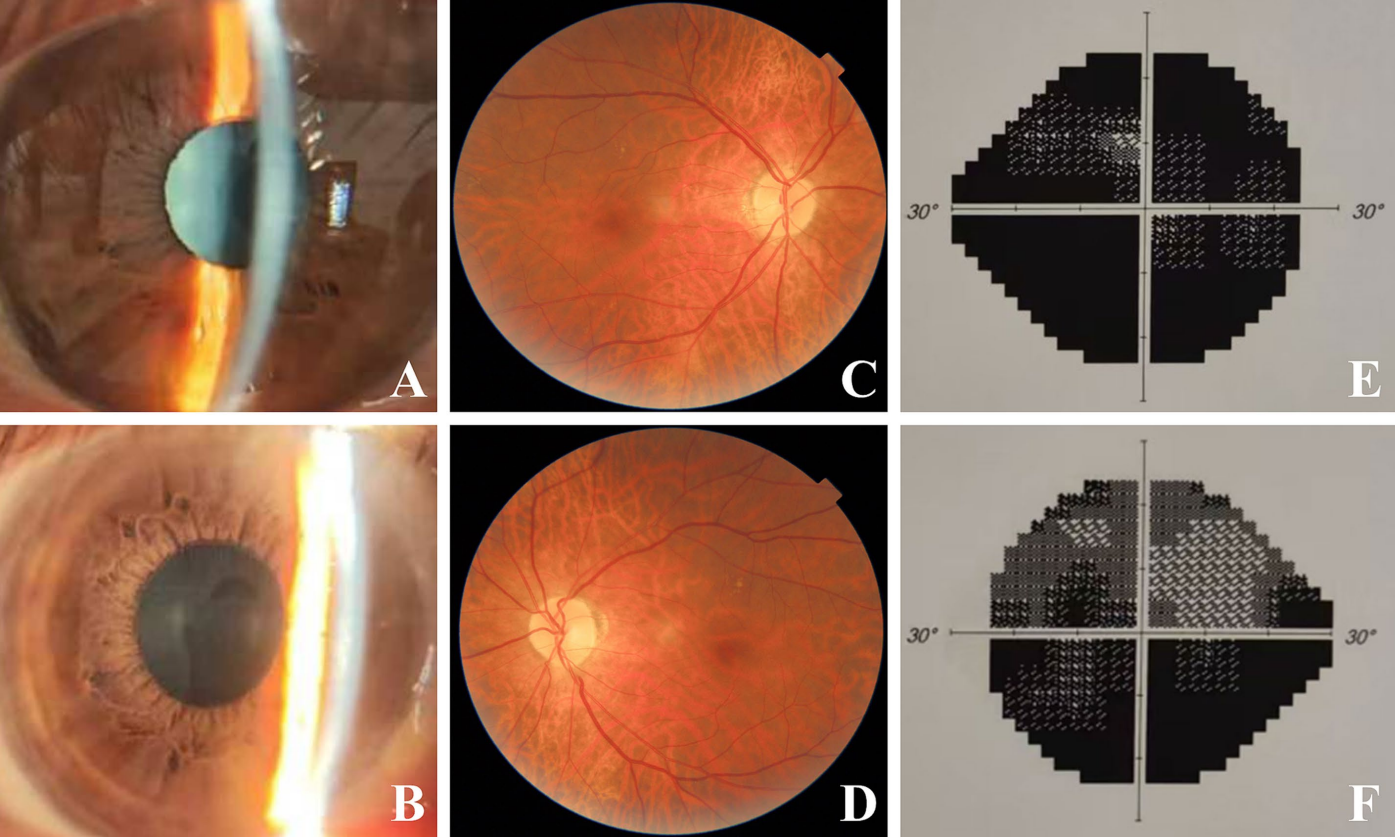
Binocular images during follow-up. Two months later after discontinuing of amiodarone, vortex keratopathy of right **(A)** and left **(B)** eye regressed absolutely. Five months after discontinuing of amiodarone, complete resolution of papilledema and hemorrhages were observed in right **(C)** and left eye **(D)**. Slight progression of visual field defect was observed in right **(E)** and left **(F)** eye.

Upon admission, systemic examinations were conducted. Laboratory findings indicated an erythrocyte sedimentation rate of 2.0 mm/h (reference range: 0–21.00 mm/h), C-reactive protein level of 4.68 mg/L (0–5.00 mg/L), international normalized ratio of 1.03 (0.75–1.10), antistreptolysin O titer of 33.3 IU/mL (0–116.0 IU/mL), and rheumatoid factor of less than 20 IU/mL (0–20 IU/mL). Tests for tuberculin, hepatitis markers, toxoplasma, cytomegalovirus, herpes simplex virus (HSV), varicella zoster virus (VZV), human immunodeficiency virus (HIV), syphilis, aquaporin-4 antibodies (NMO), and myelin oligodendrocyte glycoprotein antibodies (MOG) were all negative. Both cervical artery ultrasonography and a non-contrast brain CT scan yielded normal results. Despite empirical treatment with 500 mg/day intravenous methylprednisolone for three days, the patient’s visual acuity did not show significant improvement. The patient had taking amiodarone therapy for four months. Cardiology was consulted. A diagnosis of amiodarone induced optic neuropathy and keratopathy was made. Amiodarone was discontinued with the consent of the patient’s cardiologists, and corticosteroid treatment was gradually tapered. After two months, the corneal deposits resolved (Figures 2A,B), and BCVA was 0.1 in the right eye and 0.4 in the left eye. However, disc swelling and peridisk hemorrhage persisted in both eyes. A follow-up examination at 5 months demonstrated complete resolution of optic disc edema (Figures 2C,D) and slight progression of visual field defect in both eyes (Figures 2E,F). After one year of follow-up, BCVA and visual field remained stable, and the papilledema had fully regressed without recurrence. The patient’s diagnosis with the relevant data about the care and follow-up was summarized in Table 1.

**Table 1:** Timeline of the patient’s care and follow-up.

Time point	Key events and interventions	Clinical outcomes and examination data
November 2023	Admission for bilateral blurred vision (20 days) IV methylprednisolone 500 mg/day × 3d	BCVA: 0.08 (OD), 0.5 (OS) Corneal deposits (Figures 1A,B) Optic disc edema with peripapillary hemorrhage (Figures 1C,D) Visual field defects (Figures 1E,F)
December 2023	Amiodarone discontinued (cardiology consult) corticosteroid tapering initiated	BCVA: 0.1 (OD), 0.4 (OS) Corneal deposits resolved (Figures 2A,B) Persistent optic disc edema
March 2024	5-month follow-up	Optic disc edema resolved (Figures 2C,D) Slight progression of visual field defects (Figures 2E,F)
November 2024	1-year follow-up	Stable BCVA: 0.1 (OD), 0.4 (OS) No recurrence of optic disc edema

BCVA, best-corrected visual acuity; IV, intravenous; OD, right eye, OS, left eye.

## Discussion

Amiodarone is increasingly utilized as an effective antiarrhythmic medication. However, due to its high lipophilicity, low bioavailability and prolonged half-life, various systemic and ocular complications have emerged. Desethyl-amiodarone, the primary metabolite of amiodarone, exhibits identical pharmacological effects to the parent compound. Amiodarone and its active metabolite form a complex with lipid substrates of lysozyme, leading to deposition in lysosomal inclusion bodies (11). These lamellar bodies had been observed in nearly all ocular tissues, including the cornea, crystalline epithelium, retina, and optic nerve, causing various amiodarone-induced ocular tissue complications (12). Among these complications, amiodarone-induced keratopathy and optic neuropathy represent two clinically significant ocular manifestations. While keratopathy is the most prevalent (affecting 70–100% of long-term users), optic neuropathy, though rare (0.36–2% incidence), may lead to irreversible vision loss, underscoring the need for vigilant monitoring.

## Keratopathy

Amiodarone-induced ocular lesions were firstly described in the 1960s, with vortex keratopathy being the most prevalent manifestation. The formation of corneal deposits may be due to the secretion of amiodarone and its active metabolites in tears into the tear film. With the increase of drug concentration in tear film and prolonged exposure to ultraviolet light, the drug-lipid complex is deposited in the corneal tissue (13, 14).

Vortex keratopathy is characterized by a distinctive, linear, branching pattern of grayish or golden-brown deposits located in the lower two-thirds of the corneal epithelium. One hypothesis posits that the predominant horizontal line in this region of the cornea aligns with the lid closure line, with deposits formation due to drug concentration in the tears (15–17). Alternatively, it is suggested that these lines arise from the centripetal migration of epithelial cells (15, 18). Keratopathy is categorized into four stages based on the extent of lesion involvement through slitlamp biomicroscopy (15). Grade I is characterized by a ‘dusting’ of the cornea at the inferior pupillary margin in the midperiphery; Grade II presents with deposits arranged in a linear pattern extending from the inferior pupillary margin toward the limbus; Grade III is marked by an increased number of branching patterns in the inferior pupillary area extending into the visual axis; Grade IV is distinguished by the presence of additional clumps of deposits compared to grade III. These four grades represent a sequential progression and are associated with the dosage and duration of amiodarone therapy. The examination of all corneal layers by confocal microscopy has revealed that amiodarone keratopathy is not confined to the epithelium, but also affects the entire corneal structure, including the Bowman’s layer, stroma, and endothelium (19, 20).

Keratopathy can develop as early as 6 days following the initiation medication, although it typically manifests within 1 to 3 months (18). The severity of keratopathy correlates with both the daily dosage and the duration of amiodarone use. A higher total intake is associated with more advanced keratopathy (21, 22). Dovie et al. (23) reported a case who took 100 mg of amiodarone daily for 6 years without developing keratopathy. However, keratopathy appeared after 6 weeks when the dosage was increased from 100 mg/d to 300 mg/d. Another report indicated that grade I keratopathy was observed after 5 months of taking 400 mg of amiodarone daily, grade II keratopathy was noted at a 12-month follow up, and grade IV keratopathy emerged at a 40-month follow-up (24).

Amiodarone-induced corneal deposits are generally bilateral and symmetrical (3, 25), although some exceptional cases have been documented. Chilov et al. (13) reported a unilateral case of amiodarone keratopathy alongside corneal dysplasia in the contralateral eye, suggesting that the rapid epithelial cell turnover associated with corneal dysplasia prevents the drug-lipid complex from adhering to the corneal tissue, thereby inhibiting the development of keratopathy. Bhatt and Ramaesh (26) documented another case of unilateral lesions, where a contact lens was present on the contralateral eye. The authors suggested that the contact lens inhibited the interaction between ultraviolet light and concentrated amiodarone, thereby preventing keratopathy in the opposite eye. Another research indicated that keratopathy in donor corneas resolves entirely within two weeks following transplantation. This rapid resolution may be attributed to either the redistribution of the drug from the donor tissue into a drug-free reservoir or the swift repopulation of the donor cornea by recipient-derived epithelium (27). Another study reported that the recipient was administered amiodarone after a corneal transplant, keratopathy manifested in one eye, while no significant changes were observed in the donor cornea. A possible reason was that disruption at the graft-host interface may impede the centripetal migration of deposit-laden epithelial cells forming at the limbus (28).

Corneal deposits rarely lead to visual loss, yet, some patients experience photosensitivity, colored halos, and glare (25, 29). In case that more severe adverse reactions such as vision loss occur, discontinuation of the drug may be considered, with the keratopathy potentially resolving completely within 3 to 20 months thereafter (5, 30). Our patient did not experience any obvious foreign body sensation, photophobia and other symptoms in either eye. Consequently, the keratopathy was not subjected to any specific treatment; instead, the medication was discontinued for observation. Expectedly, the corneal pigmentation completely resolved following a two-month period of drug withdrawal.

## Optic neuropathy

AAON is an uncommon condition with an incidence of 0.36–2%. The exact mechanism of optic neuropathy remains to be determined, yet one potential hypothesis suggests that lipid inclusion body deposition in optic nerve axons may impede axonal blood flow, leading to optic disc edema (31). Vision loss in AAON patients ranges from mild and reversible to severe and permanent (32, 33).

Patients on amiodarone usually have risk factors for vascular disease, which is the primary cause of non-arteritic anterior ischemic optic neuropathy (NAION), leading to frequent misdiagnosis of AAON as NAION due to their similar clinical features. However, AAON and NAION differ in treatment and prognosis, necessitating careful differentiation.

Previous studies have documented the clinical manifestations of AAON. Macaluso et al. (34) analyzed 73 AAON patients, identifying their clinical characteristics as insidious onset, gradual progression, bilateral visual impairment, and optic disc swelling. A study by Passman et al. (35) involving 296 AAON patients showed the average duration of amiodarone use prior to vision loss was 9 months. They reported that 44% of patients experienced hidden onset, one-third exhibited no obvious symptoms, and 85% presented with optic disc edema. Nevertheless, NAION is marked by sudden, one-sided disc swelling accompanied by hemorrhage, typically resolving within several weeks (34, 36).

Purvin et al. (36) reported that 14 out of 19 patients with AAON had bilateral disc edema, while Johnson et al. (37) showed that 43 out of 55 patients experienced binocular disc edema, indicating that binocular disease was relatively common in AAON, accounting for about 80% of cases. NAION typically manifests as a unilateral condition. In a prospective study including 388 NAION patients, 18% of the cases experienced the second eye involvement within one year, increasing to 23% within two years (38).

AAON is more frequently observed in males. Johnson et al. (37) reported that 87% of the 55 patients diagnosed with AAON were male, and Cheng’s study (39) indicated that males treated with amiodarone had nearly a threefold increased risk of developing optic neuropathy compared to females. In contrast, NAION does not exhibit a sex preference.

Optic disc edema can persist for 1–8 months in AAON cases, with a median duration of approximately 3 months (37). Conversely, optic disc swelling in NAION typically resolves within 2 to 6 weeks. The prolonged half-life of amiodarone, which can be up to 100 days, may account for the extended duration of optic nerve swelling in AAON. Furthermore, NAION occurs in patients with crowded small optic disc most commonly, AAON is not associated with any specific cup-disc ratio (40–43). Based on these distinguishing features, the diagnostic criteria for AAON are delineated in the literature as follows: (1) a documented history of amiodarone use; (2) gradual onset; (3) slow progression; (4) bilateral vision defect; and (5) optic disc edema. Clinicians should be alert to the possibility of AAON in patients who do not present with the typically small, crowded optic disc associated with NAION (4, 44). The characteristics of our case aligned with these criteria, thereby corroborating the diagnosis of AAON.

When evaluating patients undergoing amiodarone therapy who develop optic disc swelling or optic neuropathy, it is crucial to consider that these patients often have significant cardiovascular conditions. Therefore, it is prudent to consult the cardiologist regarding the potential discontinuation or dosage reduction of amiodarone. Prognostic outcomes following the cessation of amiodarone vary according to several case studies. Passman et al. (37) reported that 58% of the patients experienced visual improvement, while 21% showed no change, and another 21% suffered further visual deterioration after discontinuing amiodarone. The study by Purvin et al. (39) showed nearly half of the 14 patients exhibited decreased vision during follow-up.

In the present case, although the BCVA in the right eye improved slightly after discontinuation of amiodarone, the damage to the visual field in both eyes was further aggravated. It is considered that the progressive visual field deficiency may be attributed to the underlying risk factors predisposing to secondary optic disc ischemia or the direct toxic effects of amiodarone due to given its prolonged half-life of up to 100 days. Although the pathogenesis of amiodarone-related cytotoxicity has not been fully elucidated, several studies have proposed potential mechanisms. For example, Golli-Bennour et al. (45) reported that amiodarone cytotoxicity was caused by reactive oxygen species (ROS)-related oxidative stress, another report from Bayrakçeken et al. (46) showed that ATP can antagonize amiodarone-caused oxidative damage, it is known that ATP is involved in the synthesis of antioxidants scavenging and clearing ROS. Therefore, we believe that antioxidative treatment in the future may be beneficial in preventing amiodarone-induced optic neuropathy.

## Conclusion

In conclusion, amiodarone-related ocular conditions are frequently encountered in clinical practice, with keratopathy being the most prevalent due to its typical clinical manifestations, which facilitate straightforward diagnosis. However, the incidence of AAON is relatively low, and its onset is insidious. The clinical diagnosis of AAON is challenging due to its similarities with NAION and its unclear pathogenesis. Consequently, further prospective studies are warranted to elucidate the potential pathophysiological and pathogenic mechanisms of optic neuropathy associated with amiodarone, thereby enhancing our understanding of AAON.

## Data Availability

The original contributions presented in the study are included in the article/supplementary material, further inquiries can be directed to the corresponding author/s.
